# Comparison of psychological distress, loneliness, and social network structure between Ukrainian war refugees and Polish citizens: a cross-sectional study in Wroclaw

**DOI:** 10.3389/fpubh.2025.1621003

**Published:** 2025-07-15

**Authors:** Arkadiusz Jaworski, Michał Szyszka, Daria Sosińska, Nela Tomaszewicz, Romana Bednarchuk, Kinga Kulawik, Patryk Piotrowski

**Affiliations:** ^1^Department of Psychiatry, Wroclaw Medical University, Wroclaw, Poland; ^2^Department of Psychiatry, Medical University of Warsaw, Warsaw, Poland

**Keywords:** depression, war migrants, war, Ukraine, Poland, R-UCLA, GHQ-28, general health questionnaire-28

## Abstract

**Background:**

The ongoing war in Ukraine has led to widespread displacement and a growing mental health burden among refugees. Understanding the associations between psychological distress, loneliness, and social networks among Ukrainian war migrants is essential for designing effective support strategies.

**Methods:**

A cross-sectional survey was conducted between October 2022 and January 2023, including 209 participants (68 Ukrainian refugees and 141 Polish citizens). Standardized instruments were used: the General Health Questionnaire-28 (GHQ-28) to assess psychological distress, the Revised UCLA Loneliness Scale (R-UCLA), and the Courage Social Network Index (CSNI). Statistical analyses included t-tests, chi-square tests, Spearman correlation, and multiple linear regression to assess group differences and associations among variables.

**Results:**

Ukrainian refugees reported significantly higher levels of psychological distress, particularly in somatic symptoms and anxiety/insomnia, compared to Polish citizens. No significant differences in loneliness levels were observed between groups. Refugees demonstrated stronger social networks, particularly in family connections. Psychological distress and depressive symptoms were positively associated with higher loneliness, while stronger social networks were negatively associated with loneliness. In multiple regression models, refugee status, greater loneliness, younger age, and psychiatric history significantly predicted higher psychological distress. Conversely, higher psychological distress, older age, and weaker social networks predicted greater loneliness.

**Conclusion:**

The findings underscore the heightened psychological burden among Ukrainian refugees and suggest a protective role of family-based social networks against loneliness. Interventions should prioritize culturally sensitive mental health services, facilitate access to care, and leverage familial support systems. Longitudinal studies are warranted to examine changes in psychological well-being over time.

## 1 Introduction

Conflicts and crises are prevalent global issue that affect individuals’ mental and physical well-being. Societies worldwide are rebuilding economic and healthcare systems, while many individuals continue to struggle with long-term mental health consequences of the COVID−19 pandemic (1, 2). In the European context, the ongoing war in Ukraine represents another crisis with profound implications for both the country itself and host nations, including Poland, where refugees seek safety. By early 2023, 997,737 Ukrainian immigrants were registered in Poland (3). It is well established that war leads to psychological distress, and studies have demonstrated a causal relationship with the occurrence of mental disorders. Research has shown that the prevalence of mental disorders in conflict settings can reach 22.1% (4). Importantly, the mental health consequences of armed conflict extend beyond those directly involved in the war (5). Immigrants face multiple challenges, including cultural adjustment and loneliness (6, 7). Furthermore, symptoms of post-traumatic stress disorder (PTSD) may affect up to 50% of refugee populations, with war-related trauma, prolonged uncertainty, perceived loss of control, and experiences of discrimination identified as major contributing factors to the development of symptoms (8).

Loneliness, often described as a silent epidemic in modern society, has profound effects on mental and physical health. War refugees are often forced to leave behind relatives and friends, increasing their vulnerability to isolation. However, even individuals without such experiences may self-isolate. Studies indicate that approximately one-third of people in industrialized countries experience loneliness (9). Loneliness is associated with depression, anxiety, suicidal ideation, and increased risk of addiction and substance use disorders (10, 11).

Numerous studies, including those conducted by international organizations, support the view that psychological factors are inseparable from overall health (12). Social isolation and loneliness are common sources of chronic stress in adults, affecting the vagal system, immune function, and oxidative stress response. Population-based studies have identified social isolation as a risk factor for cardiovascular disease and a predictor of premature mortality (9, 13). Moreover, subjective feelings of loneliness impair cognitive function in older adults and may predict dementia onset (14). Evidence also links social isolation to altered eating behaviors and adverse metabolic outcomes. Loneliness, especially when accompanied by mental health symptoms, has been shown to influence brain reactivity to food cues, including cravings and reward-based eating (15).

For refugees, the experience of displacement may intensify social isolation, further hindering the adaptation process. As Ukrainian immigrants integrate into host communities, social networks begin to form. The structure of social networks, defined by the quality and quantity of interpersonal relationships, plays a critical role in buffering individuals against loneliness (16). These networks serve to protect against both psychological and somatic consequences of loneliness (17). Social integration may be particularly important given that a substantial proportion of refugees are older adults who are significantly burdened by cardiovascular, gastrointestinal, respiratory, musculoskeletal, and genitourinary diseases (18).

The importance of social contacts in reducing stress and protecting mental health has already been previously presented and discussed in the literature through conceptual models such as the stress-buffering hypothesis and social capital theory. Cohen and Wills (19) connected the stress-buffering hypothesis with the role of social support, particularly through the buffering model, in which social support mitigates the negative psychological effects of stress. Social support can take the form of emotional support, informational support, social companionship, and instrumental support. Numerous studies have confirmed the protective role of social support in alleviating psychiatric symptoms among refugees, especially those related to PTSD, anxiety, and depression (20–22). A concept closely related to social support is cognitive social capital (23). Social capital is an umbrella term encompassing a broad range of social resources (24–28). In the context of this study, social capital refers to the social resources available to an individual through relationships and community ties, which can support well-being and resilience (23, 24). These resources include both subjective perceptions, such as trust, a sense of belonging, and mutual reciprocity, and objective features, such as social networks, shared norms, and participation in collective life. Two key dimensions of social capital are distinguished: cognitive social capital, which reflects subjective perceptions such as trust, shared values, and sense of belonging, and structural social capital, which refers to the objective presence of social ties, group memberships, and participation in social networks (23, 29). Additionally, social capital can be categorized into bonding (close ties within homogeneous groups such as family or coethnic peers), bridging (connections between socially diverse groups), and linking (vertical relationships with institutions and power structures), each serving different roles in adaptation and mental health (24). Cognitive and structural forms of social capital are associated with lower levels of depression, anxiety, and posttraumatic stress symptoms among refugees, particularly when social resources enhance feelings of security, trust, and social belonging (23, 24, 30). In particular, bonding capital - strong ties within coethnic or familial groups - can buffer against emotional isolation and promote a sense of identity and safety in the early phases of resettlement. Meanwhile, bridging and linking capital may support access to host community resources, foster institutional trust, and reduce structural barriers to care and integration (31–33). A growing body of research also highlights the effectiveness of social capital-based mental health interventions, which aim to strengthen interpersonal relationships, rebuild trust, and promote social inclusion. Systematic reviews show that such interventions may reduce symptoms of psychological distress, improve emotional well-being, and promote social functioning in refugee populations (30, 31). By targeting social connectedness directly, these programs address not only individual symptoms but also the broader social determinants of refugee mental health.

Understanding how social networks manifest among Ukrainians and Poles offers valuable insight into the mechanisms of social integration and support, and helps clarify how psychosocial factors relate to psychological distress in both populations. To the best of our knowledge, this is the first study to directly compare psychological distress, loneliness levels, and social network structure between Ukrainian war refugees and Polish citizens. Although research on refugee mental health has expanded in recent years, few studies have investigated these psychosocial dimensions within a comparative framework conducted in the same host country. This study addresses a significant gap in the literature by simultaneously examining these variables in a cross-sectional sample recruited in Wroclaw - one of Poland’s largest urban centers and a major hub for Ukrainian refugees, currently hosting approximately 4.5% of individuals under temporary protection (3).

The main aim is to identify areas for targeted intervention and support for refugees during crises of this nature. We hypothesize that the prevalence of depression and anxiety differs between the studied groups, with lower levels expected among Polish citizens. In addition, we expect that social networks are less developed among Ukrainian participants and that their emotional distress is higher, reflecting the complex mental health consequences of war-related displacement (34).

The study aims to:

1. Compare the level of psychological distress, loneliness and social network between Ukrainian and Polish citizens during the ongoing war and migration crisis.
2. Evaluate the associations between loneliness, social network structure, and mental well-being in both populations.
3. Identify key sociodemographic and psychosocial factors associated with psychological distress and loneliness.

## 2 Materials and methods

### 2.1 Study design

We conducted this cross-sectional study from October 2022 to January 2023. We obtained approval from the Bioethics Committee at Wroclaw Medical University on 27 October 2022 (No. KB-777/2022). The study was part of a larger project, Not Alone in the Crowd, which addressed the issue of loneliness and was funded by the Student Activity Fund (FAST) provided by the City of Wroclaw. We employed a convenience sampling strategy. Our interviewers recruited participants in public locations across Wroclaw, including three shopping malls, two refugee assistance centers, the Department for Foreigners at the Lower Silesian Voivodeship Office, a university campus, two senior citizens’ clubs, and during a university lecture that formed part of the Not Alone in the Crowd project. We selected these sites based on their anticipated high traffic of both Ukrainian refugees and Polish citizens living in Wroclaw. We invited individuals at these sites to complete a paper-based questionnaire. In addition to direct recruitment at designated locations, some participants were recruited using the snowball sampling method, whereby initial respondents referred others within their social networks. All participants provided informed consent, which we included in the survey instructions. The researchers were on site and addressed all questions regarding the items in the psychometric scales in cases of any doubts.

### 2.2 Participants

The study included adult participants aged 18 years or older. The sample consisted of two groups: Polish citizens residing in Wroclaw and Ukrainian citizens who had lived in Ukraine prior to the outbreak of the war and had subsequently sought refuge in Wroclaw. The inclusion and exclusion criteria were defined separately for each group:

Polish citizens:

- Inclusion criteria:

o age ≥18,
o Polish citizenship,
o current residency in Wroclaw

- Exclusion criteria:

o age <18;
o inability to provide informed consent;
o insufficient understanding of study procedures despite clarification.

Ukrainian war refugees:

- Inclusion criteria:

o age ≥18,
o Ukrainian citizenship,
o prior permanent residency in Ukraine before February 2022,
o relocation to Poland as a result of the war

- Exclusion criteria:

o age <18;
o inability to provide informed consent;
o insufficient understanding of study procedures despite clarification;
o migration to Poland before the war for reasons unrelated to the conflict (e.g., employment or education).

To ensure accurate identification of war refugee status, a Ukrainian-speaking co-author (RB) actively participated in participant recruitment within refugee-focused locations. During initial contact, she conducted a brief conversation in Ukrainian to confirm the participant’s background and refugee status. Following confirmation, she provided all study-related information both in writing and, if needed, orally in Ukrainian to ensure full comprehension and support informed consent. To minimize selection bias, interviewers were instructed to recruit participants with the greatest possible diversity in terms of visually identifiable characteristics such as age, gender, and ethnicity. They consciously avoided preferential selection based on appearance or perceived approachability. Additionally, interviewers changed their position within each recruitment site at regular intervals (e.g., within shopping malls or refugee centers) to reduce environmental and temporal biases. Due to the nature of the selected sites, including high foot traffic, dynamic interpersonal interactions, and variation in timing and staffing across locations, we did not systematically collect data on response rates.

### 2.3 Instruments

We distributed the questionnaires in both Ukrainian and Polish language versions. A native Ukrainian-speaking author (RB) translated the Polish version of the questionnaire using psychometric tools, and an independent native speaker performed the back-translation. The survey collected data on sociodemographic characteristics, including age, sex, place of residence, citizenship, war refugee status, education level, occupation, receipt of social benefits, and personal or family psychiatric history. The following variables were assessed using binary (yes/no) questions: citizenship, war refugee status, receipt of social benefits, personal psychiatric history, and family psychiatric history. Other variables were assessed using categorical or continuous response formats: age (open-ended), sex (categorical), education level (primary/secondary/higher), and occupation (employed/unemployed). We also administered three standardized instruments: the GHQ-28 (35), the Revised UCLA Loneliness Scale (36), and the Courage Social Network Index (37).

The General Health Questionnaire (GHQ-28) is a widely recognized psychometric tool for screening and assessing current nonpsychotic psychopathology in adults (38). It primarily reflects symptoms of depression and related affective disturbances. The scale has been validated for use in Polish occupational health settings (35). It includes 28 items rated on a 4-point Likert scale, yielding a total score ranging from 0 to 84, with higher scores indicating greater psychological distress. A score of 24 or above is considered clinically significant. The GHQ-28 comprises four subscales, each containing seven items: Somatic Symptoms (Subscale A), Anxiety and Insomnia (Subscale B), Social Dysfunction (Subscale C), and Severe Depression (Subscale D). In the current sample, the GHQ-28 demonstrated high internal consistency (Cronbach’s *α* = 0.93). The reliability of individual subscales was also satisfactory: α = 0.82 for Somatic Symptoms, α = 0.85 for Anxiety and Insomnia, α = 0.87 for Social Dysfunction, and α = 0.90 for Severe Depression.

The Revised UCLA Loneliness Scale (R-UCLA) is based on the original version developed by Russel et al. (39). For this study, we used the Polish version validated by Kwiatkowska et al. (36). The scale consists of 20 items rated on a 4-point Likert scale: 1 = never, 2 = rarely, 3 = sometimes, and 4 = often. Following the recommendation of Kwiatkowska et al., we excluded item 4 (“I do not feel alone”) from the analysis due to its direct wording and poor correlation with the total score. As a result, the maximum total score was 76. The R-UCLA includes three conceptually distinct subscales: Belonging and Affiliation, Intimate Others, and Social Others, each contributing to the total loneliness score. We calculated the total score as the sum of responses (excluding item 4), yielding a possible range of 16 to 76 points. For analytical purposes, we categorized loneliness levels into four groups: low (16–30 points), moderate (31–45), moderately high (46–60), and high (61–76) (40, 41). Cronbach’s alpha indicated high internal consistency for the total R-UCLA score (*α* = 0.89) and acceptable reliability for the three subscales (α = 0.70, 0.83, and 0.80, respectively).

The Courage Social Network Index (CSNI) is a multidimensional instrument developed to assess both the structure and function of individuals’ social networks. This tool was validated in a large European study, including a Polish population, and showed good reliability and content validity (37). It evaluates three key functional dimensions across each structural component: the closeness of social ties, the perceived availability of general support, and the frequency of face-to-face contact. There are 8 questions in each element of the social network corresponding to 5 structural components (spouse or partner, parents, other family members, neighbors, friends and co-workers). The score was obtained by the Item Response Theory procedure and the results were interpreted as social network saturation, where 0 indicated the lowest level of saturation and 100 the highest.

The CSNI presented satisfactory internal consistency (*α* = 0.77 overall; α = 0.92, α = 0.86, α = 0.82, α = 0.76, α = 0.80, respectively, for five structural components).

Internal consistency of all instruments and their subscales was evaluated separately for the Ukrainian versions used to assess the refugee group and the Polish versions administered to the Polish residents of Wroclaw. The reliability of all tools was satisfactory across both language groups. Cronbach’s alpha coefficients are summarized in Table 1.

**Table 1:** Internal consistency (Cronbach’s alpha) for each instrument by language group and combined samples.

Instrument	Ukrainian version (α)	Polish version (α)	Combined (α)
GHQ-28	0.91	0.94	0.93
Somatic symptoms	0.83	0.78	0.82
Anxiety/insomnia	0.83	0.87	0.85
Social dysfunction	0.86	0.88	0.87
Severe depression	0.82	0.91	0.90
R-UCLA	0.89	0.90	0.89
Intimate others	0.80	0.85	0.83
Social others	0.83	0.78	0.80
Belonging and affiliation	0.66	0.73	0.70
C-SNI	0.83	0.73	0.77
Spouse/partner	0.92	0.93	0.92
Parents	0.85	0.86	0.86
Other family members	0.77	0.85	0.82
Neighbors	0.75	0.77	0.76
Friends and co-workers	0.81	0.80	0.80

### 2.4 Statistical analysis

Descriptive statistics are reported as means ± standard deviations (SD), absolute frequencies, and percentages. Little’s MCAR test indicated that missing data were missing completely at random (*p* = 0.411). Therefore, missing values were imputed using the Expectation–Maximization (EM) algorithm, applied at the subscale level for each questionnaire. Sum scores were calculated to evaluate general health (GHQ-28), loneliness (R-UCLA), and weighted social network saturation (COURAGE-Social Network Index; see below). The Shapiro–Wilk test was used to determine the normality of data distribution, and the Levene’s test was applied to assess homogeneity of variances. A chi-square test (or Fisher’s exact test, where appropriate) with Bonferroni correction was used to compare categorical variables (42). Differences between groups were tested using the Kruskal–Wallis test and one-way analysis of variance (ANOVA) when more than two groups were compared. For two-group comparisons, the independent samples t-test or Mann–Whitney U-test was used, as appropriate. Multiple linear regression was used to analyze potential associations between dependent variables: depressive symptoms (GHQ-28), loneliness (R-UCLA), and multiple independent variables. Dichotomous variables were dummy coded. The statistical significance level was set at *p* < 0.05. All analyses were performed using IBM SPSS Statistics, Version 27.

We applied Item Response Theory (IRT) modeling to evaluate latent traits within individuals across five distinct components of the COURAGE-SNI framework: spouse/partner, parents, other family members, neighbors, friends/co-workers. Following the methodology proposed by Zawisza et al. (37), we conducted all analyses in R (version 4.2.2),^1^ using the mirt package (version 1.41) for IRT modeling and tidyverse package (version 2.0.0) for data manipulation. To estimate individual latent trait positions, we used the Generalized Partial Credit Model (GPCM), developed by Muraki (43). This model produced factor scores that captured each participant’s placement along the latent trait continuum. We then standardized these scores within each structural component to compute a global social network score, expressed on a 0 to 100% scale. Consistent with Zawisza et al.’s findings, we observed that varying structural components contribute differentially to the assessment of the total score for the COURAGE-SNI. Consequently, we derived Total Component Information (TCI) from the summary of values provided by the Item Information Function (IIF) (44) for items across all components. These TCIs served as weights in the calculation of the weighted total Social Network Score (WTSNS), thereby facilitating the determination of the COURAGE-SNI total score for each study participant.

## 3 Results

### 3.1 Participants’ characteristics

The survey included 209 participants: 68 Ukrainian refugees and 141 Polish citizens residing in Wroclaw. Women constituted the majority of the sample (*n* = 149; 71.29%). The mean age of all participants was 40.22 ± 18.76 years. Ukrainian refugees were significantly older than Polish citizens (44.01 ± 15.58 vs. 38.38 ± 19.91 years; Mann–Whitney U test: Z = −2.925, *p* = 0.003). Regarding education, 95 participants (45.45%) reported having higher education. Tables 2, 3 present the demographic characteristics and comparisons on depression and loneliness measures for Ukrainian refugees and Polish citizens, respectively.

**Table 2:** Sociodemographic characteristics of Ukrainian refugees—associations with GHQ-28 and R-UCLA total scores.

Parameter	N	%	GHQ-28 total score			GHQ-28	R-UCLA total score			R-UCLA
			M	Median	SD	t/F	M	Median	SD	t/F
Gender										
Female	52	76.47%	34.01	33.00	12.32	*t* = 1.579 *p* = 0.119	36.29	33.00	10.16	*t* = −0.244 *p =* 0.808
Male	16	23.53%	28.56	30.00	11.15		36.99	36.50	9.49	
Total	68	100.00%	32.73	32.00	12.20		36.45	34.34	9.94	
Education										
Primary	2	2.94%	27.50	27.50	20.51	*F* = 0.245 *p* = 0.783	34.65	34.65	2.37	*F* = 0.126 *p* = 0.882
Secondary	25	36.76%	33.56	31.00	12.93		37.19	36.00	9.99	
Higher	41	60.29%	32.47	32.00	11.68		36.09	33.00	10.24	
Employment status										
Employed	32	47.06%	30.63	30.00	13.02	*t* = −1.348 *p* = 0.182	35.62	32.99	9.79	*t* = −0.647 *p* = 0.520
Unemployed	36	52.94%	34.60	34.00	11.26		37.19	36.00	10.16	
Collecting social benefits										
Yes	21	30.88%	33.05	35.00	12.92	*t* = 0.146 *p* = 0.884	35.15	34.00	8.18	*t* = −0.719 *p* = 0.475
No	47	69.12%	32.58	32.00	12.00		37.03	36.00	10.67	
Psychiatric history										
Yes	2	2.94%	51.00	51.00	16.97	*t* = 2.212 ***p* = 0.030**	45.37	45.37	1.93	*t* = 1.293 *p* = 0.200
No	66	97.06%	32.17	31.50	11.76		36.18	34.00	9.97	
Psychiatric history in family										
Yes	1	1.47%	38.00	38.00	N/A	*t* = 0.433 *p* = 0.667	51.00	51.00	N/A	*t* = 1.487 *p* = 0.142
No	67	98.53%	32.65	32.00	12.27		36.24	34.00	9.86	

t, Student’s *t*-test; F, ANOVA’s test; *p*, statistical significance; significant differences are marked in bold. Average age of Ukrainian refugees was 44 years old (Median = 41; SD = 15.58). For female participants, the average age was 45 years (Median = 45; SD = 14.64), while for males, the average score was 41 years (Median = 37; SD = 18.53).

**Table 3:** Sociodemographic characteristics of Polish citizens of Wroclaw—associations with GHQ-28 and R-UCLA total scores.

Parameter	N	%	GHQ-28 total score			GHQ-28	R-UCLA total score			R-UCLA
			M	Median	SD	t/Z/H	M	Median	SD	t/Z/H
Gender										
Female	97	68.79%	27.72	24.00	14.58	*t* = 0.403 *p* = 0.688	36.21	34.00	10.07	*t* = −0.083 *p* = 0.934
Male	44	31.21%	26.65	22.50	14.81		36.36	35.00	9.63	
Total	141	100.00%	27.39	23.80	14.61		36.26	35.00	9.90	
Education										
Primary	8	5.67%	19.97	21.50	6.89	*H* = 2.350 *p* = 0.309	36.13	35.00	7.24	*H* = 0.822 *p* = 0.663
Secondary	79	56.03%	28.85	25.00	15.88		36.74	37.00	10.03	
Higher	54	38.30%	26.34	23.00	13.17		35.57	33.00	10.16	
Employment status										
Employed	88	62.41%	28.80	25.00	14.61	*Z* = −1.967 ***p* = 0.049**	35.63	34.00	9.87	*Z* = −1.031 *p* = 0.303
Unemployed	53	37.59%	25.03	21.00	14.42		37.32	37.00	9.96	
Collecting social benefits										
Yes	16	11.35%	22.92	18.00	13.32	*Z* = −1.805 *p* = 0.071	35.75	33.00	8.18	*Z* = −0.127 *p* = 0.899
No	125	88.65%	27.96	24.00	14.71		36.33	35.00	10.13	
Psychiatric history										
Yes	32	22.70%	37.97	36.50	18.37	*Z* = −4.141 ***p* = <0.001**	42.06	41.00	10.91	*t* = 3.961 ***p* = < 0.001**
No	109	77.30%	24.28	22.00	11.69		34.56	33.00	8.94	
Psychiatric history in family										
Yes	51	36.17%	32.18	29.00	16.82	*Z* = −3.022 ***p* = 0.003**	38.21	37.00	10.39	*t* = 1.777 *p* = 0.078
No	90	63.83%	24.67	21.50	12.49		35.15	33.00	9.49	

t, Student’s *t*-test; Z, Mann–Whitney U-test; H, Kruskal–Wallis test; *p*, statistical significance; significant differences are marked in bold. Average age of Polish citizens was 38 years old (Median = 28; SD = 19.91). For female participants, the average age was 41 years (Median = 28; SD = 21.02), while for males, the average score was 33 years (Median = 28; SD = 16.37).

With respect to sociodemographic comparisons, we found no significant differences in gender distribution between Ukrainian refugees and Polish citizens. However, the groups differed in educational attainment: 56.03% of Polish participants had completed secondary education (vs. 36.76% of Ukrainians; *p* = 0.027), while 60.29% of Ukrainian participants held higher education degrees (vs. 38.30% of Poles; *p* = 0.008). A larger proportion of Ukrainian participants were unemployed (52.94%) compared to Polish citizens (37.59%). The chi-square test indicated a significant difference (*p* = 0.035), but this result did not remain significant after Bonferroni correction (adjusted *p* = 0.071). In addition, 30.88% of Ukrainian refugees reported receiving social benefits, compared to 11.35% of Polish participants (*p* < 0.001). Polish participants more frequently reported a personal history of psychiatric disorders (22.70% vs. 2.94%, *p* < 0.001) and a family history of psychiatric conditions (36.17% vs. 1.47%, *p* < 0.001). Table 4 summarizes these between-group sociodemographic differences.

**Table 4:** Differences between Ukrainian refugees and Polish citizens of Wrocław regarding categorical variables.

Parameter	Total sample		Ukrainian refugees		Polish citizens of Wrocław		Chi-square tests		
	N	%	N	%	N	%	χ^2^	*p*-value/Bonferonii adjusted *p*-value	Cramer’s V
Gender							1.321	0.250	0.079
Female	149	71.29%	52	76.47%	97	68.79%		N/A	
Male	60	28.71%	16	23.53%	44	31.21%		N/A	
Education							9.02	**0.011**	0.208
Primary	10	4.78%	2	2.94%	8	5.67%		1.153	
Secondary	104	49.76%	25	36.76%	79	56.03%		**0.027**	
Higher	95	45.45%	41	60.29%	54	38.30%		**0.008**	
Employment status							4.422	**0.035**	0.145
Employed	120	57.42%	32	47.06%	88	62.41%		0.071	
Unemployed	89	42.58%	36	52.94%	53	37.59%		0.071	
Collecting social benefits							12.016	**<0.001**	0.24
Yes	37	17.70%	21	30.88%	16	11.35%		**<0.001**	
No	172	82.30%	47	69.12%	125	88.65%		**<0.001**	
Psychiatric history							13.142	**<0.001**	0.251
Yes	34	16.27%	2	2.94%	32	22.70%		**<0.001**	
No	175	83.73%	66	97.06%	109	77.30%		**<0.001**	
Psychiatric history in family							29.554	**<0.001**	0.376
Yes	52	24.88%	1	1.47%	51	36.17%		**<0.001**	
No	157	75.12%	67	98.53%	90	63.83%		**<0.001**	
GHQ-28 relevant distress							12.098	**<0.001**	0.241
Present	121	57.89%	51	75.00%	70	49.65%		**<0.001**	
Absent	88	42.11%	17	25.00%	71	50.35%		**<0.001**	
Level of loneliness							0.022	0.989	0.01
Low	103	49.52%	34	50.00%	69	49.29%		N/A	
Moderate	81	38.94%	26	38.24%	55	39.29%		N/A	
Moderately high	24	11.54%	8	11.76%	16	11.43%		N/A	
High	0	0.00%	0	0.00%	0	0.00%		N/A	

χ^2^, Pearson chi-square; *p*, statistical significance; significant differences are marked in bold.

### 3.2 Mental health status

Based on the GHQ-28 analysis, 121 participants (57.89%) scored above the clinical cut-off for psychological distress (Table 4). Ukrainian refugees reported significantly higher total GHQ-28 scores than Polish citizens. Among refugees, 51 individuals (75%) exceeded the clinical threshold, with a mean score of 32.73 ± 12.20 (*p* < 0.01; Tables 4, 5). In relation to GHQ-28 subscales, Ukrainian refugees scored significantly higher on Somatic Symptoms and Anxiety/Insomnia, with mean scores of 10.64 ± 4.22 and 9.88 ± 4.51, respectively (*p* < 0.001 and *p* < 0.003). We found no significant differences in GHQ-28 total scores between males and females in either group. Among Polish citizens, employed participants had significantly higher GHQ-28 scores than those unemployed (*p* = 0.049). Polish participants with a psychiatric history and those with a family history of psychiatric disorders also reported significantly higher scores (*p* < 0.001 and *p* = 0.003, respectively). In the Ukrainian refugee group, individuals with a psychiatric history scored significantly higher than those without (*p* = 0.03). Associations between GHQ-28 scores and sociodemographic characteristics are presented in Table 2 for Ukrainian refugees and in Table 3 for Polish citizens.

**Table 5:** Independent *t*-tests comparing GHQ-28, R-UCLA, and C-SNI scores between Ukrainian refugees and Polish citizens of Wroclaw.

Scale/subscale	Ukrainian refugees			Polish citizens of Wrocław			t	Cohen’s D	*p*-value (2-tailed)
	M	Median	SD	M	Median	SD			
GHQ-28	32.73	32.00	12.20	27.39	23.80	14.61	2.607	0.385	**0.01**
Somatic symptoms	10.64	10.00	4.22	7.61	7.00	3.97	5.061	0.747	**0.001**
Anxiety/insomnia	9.88	8.71	4.51	7.70	6.00	4.96	3.051	0.451	**0.003**
Social dysfunction	9.04	8.06	3.91	7.98	7.00	3.64	1.923	0.284	0.056
Severe depression	3.17	2.00	3.50	4.09	2.00	5.08	−1.525	−0.199	0.129
R-UCLA total score	36.45	34.34	9.94	36.26	35.00	9.90	0.132	0.019	0.895
Intimate others	21.72	21.00	5.84	20.92	20.00	6.18	0.889	0.131	0.375
Social others	8.06	7.00	3.20	8.11	7.00	2.76	−0.114	−0.017	0.909
Belonging and affiliation	8.03	8.00	2.56	8.53	8.00	2.75	−1.261	−0.186	0.209
C-SNI	50.71	53.19	17.95	40.88	40.46	16.63	3.9	0.576	**0.001**
Spouse/partner	51.82	54.97	38.58	44.54	56.13	43.37	1.226	0.174	0.222
Parents	42.86	51.47	30.84	48.66	55.83	30.76	−1.275	−0.188	0.204
Other family members	51.63	58.47	25.33	26.52	9.17	26.95	6.575	0.95	**0.001**
Neighbors	54.20	57.17	25.03	51.05	53.36	23.56	0.889	0.131	0.375
Friends and co-workers	48.94	52.97	23.43	55.30	57.80	19.68	−1.934	−0.303	0.056

t, Student’s *t*-test; *p*, statistical significance; significant differences at *p* ≤ 0.05 are marked in bold.

### 3.3 Level of loneliness

We found no statistically significant differences in either the categorical levels of loneliness (Table 4), the total R-UCLA score, or any of the subscale scores between Polish citizens and Ukrainian refugees (Table 5). One Polish participant had a total R-UCLA score of 19, which fell outside the defined cutoff ranges for loneliness classification. As a result, we excluded this individual from the chi-square test of independence. Among Polish citizens, those with a positive psychiatric history reported significantly higher levels of loneliness compared to those without (*p* < 0.001). Associations between R-UCLA scores and sociodemographic variables are presented in Table 2 for Ukrainian refugees and Table 3 for Polish citizens.

### 3.4 Structure of social networks

Ukrainian refugees demonstrated significantly higher mean total scores on the CSNI compared to Polish citizens (*p* = 0.001). Among the five structural components, only the “other family members” domain showed a significant difference between the groups, with refugees scoring higher than Polish participants (*p* = 0.001). We found no significant group differences in the remaining components: spouse/partner, parents, neighbors, or friends/co-workers. Table 5 presents the mean total CSNI scores and the mean scores for each of the five components in both groups.

### 3.5 Correlations

We observed a strong positive correlation between the GHQ-28 total score and the R-UCLA total score (*p* < 0.01, *r* = 0.502). The GHQ-28 total score was also significantly associated with all R-UCLA subscale scores (*p* < 0.01; *r* = 0.521, 0.389, and 0.349, respectively). Higher loneliness scores (R-UCLA total) were moderately and inversely correlated with the CSNI total score (*p* < 0.01, *r* = −0.303). Similar negative correlations were found between R-UCLA subscales and the CSNI total score (*p* < 0.01; *r* = −0.265, −0.358, and −0.195, respectively). Age was positively associated with the CSNI total score (*p* < 0.01, *r* = 0.305), and with the “Other family members” and “Neighbors” subscales (*p* < 0.01, *r* = 0.665 for both). In contrast, age was negatively correlated with the “Parents” (*p* < 0.01, *r* = −0.577) and “Friends and Co-workers” subscales (*p* < 0.05, *r* = −0.137). We also observed negative correlations between age and the GHQ-28 total score (*p* < 0.05, *r* = −0.146) and the Severe Depression subscale (*p* < 0.01, *r* = −0.185). Table 6 presents all correlation coefficients among GHQ-28, R-UCLA, and CSNI scores, as well as their associations with age, calculated across all participants. See Supplementary Tables S1, S2 in the appendix for group-specific correlations.

**Table 6:** Spearman’s rho Correlation Matrix for Age and Scores Among Scales and Subscales in All Participants (N = 209).

Parameter	Age	GHQ-28 total score	Somatic symptoms	Anxiety/insomnia	Social dysfunction	Severe depression	R-UCLA total score	Intimate others	Social others	Belonging and affiliation	C-SNI	Spouse/partner	Parents	Other family members	Neighbors
GHQ-28 total score	**−0.146** ^*^														
Somatic symptoms	−0.082	**0.803** ^**^													
Anxiety/insomnia	−0.115	**0.861** ^**^	**0.645** ^**^												
Social dysfunction	−0.057	**0.769** ^**^	**0.479** ^**^	**0.580** ^**^											
Severe depression	**−0.185** ^**^	**0.691** ^**^	**0.344** ^**^	**0.501** ^**^	**0.486** ^**^										
R-UCLA total score	−0.011	**0.502** ^**^	**0.303** ^**^	**0.426** ^**^	**0.408** ^**^	**0.555** ^**^									
Intimate others	0.027	**0.521** ^**^	**0.343** ^**^	**0.475** ^**^	**0.402** ^**^	**0.541** ^**^	**0.918** ^**^								
Social others	−0.105	**0.389** ^**^	**0.221** ^**^	**0.326** ^**^	**0.331** ^**^	**0.409** ^**^	**0.849** ^**^	**0.683** ^**^							
Belonging and affiliation	−0.022	**0.349** ^**^	**0.180** ^**^	**0.264** ^**^	**0.294** ^**^	**0.436** ^**^	**0.775** ^**^	**0.533** ^**^	**0.636** ^**^						
C-SNI	**0.305** ^**^	−0.069	0.051	−0.043	−0.112	**−0.174** ^*^	**−0.303** ^**^	**−0.265** ^**^	**−0.358** ^**^	**−0.195** ^**^					
Spouse/partner	0.009	−0.070	0.007	−0.022	**−0.159** ^*^	**−0.136** ^*^	**−0.285** ^**^	**−0.288** ^**^	**−0.306** ^**^	−0.124	**0.811** ^**^				
Parents	**−0.577** ^**^	−0.025	−0.017	−0.037	−0.064	0.009	−0.134	**−0.165** ^*^	−0.024	−0.086	0.035	0.117			
Other family members	**0.665** ^**^	0.003	0.113	−0.025	0.051	−0.081	−0.012	0.047	**−0.143** ^*^	−0.061	**0.473** ^**^	0.025	**−0.424** ^**^		
Neighbors	**0.313** ^**^	**−0.172** ^*^	−0.082	**−0.151** ^*^	**−0.175** ^*^	**−0.185** ^**^	**−0.179** ^**^	-0.099	**−0.226** ^**^	**−0.208** ^**^	**0.396** ^**^	0.067	−0.072	**0.344** ^**^	
Friends and co-workers	**−0.137** ^*^	**−0.178** ^*^	−0.117	**−0.156** ^*^	**−0.152** ^*^	**−0.173** ^*^	**−0.357** ^**^	**−0.352** ^**^	**−0.292** ^**^	**−0.212** ^**^	**0.261** ^**^	**0.156** ^*^	**0.327** ^**^	−0.112	**0.214** ^**^

The values in the table are correlation coefficients; significant are marked in bold; *Correlation is significant at the 0.05 level (2-tailed); **Correlation is significant at the 0.01 level (2-tailed).

### 3.6 Multiple linear regression

We conducted two multiple linear regression models to examine predictors of GHQ-28 and R-UCLA total scores. Dichotomous variables were dummy-coded as follows: citizenship (0 = Polish citizen of Wroclaw, 1 = Ukrainian war migrant) and psychiatric history (0 = no psychiatric history, 1 = presence of psychiatric history). Both models were statistically significant based on F-tests [GHQ-28: *F*(5, 203) = 27.45, *p* < 0.001; R-UCLA: F(5, 203) = 26.12, *p* < 0.001], indicating that the included predictors explained a meaningful proportion of variance in the outcomes. The GHQ-28 model explained 38.9% of the variance (Adjusted R^2^ = 0.389), while the R-UCLA model accounted for 37.6% (Adjusted R^2^ = 0.376), suggesting acceptable model fit. In the GHQ-28 model, higher scores were associated with being a war migrant (*β* = 0.225), greater loneliness (*β* = 0.516), younger age (*β* = −0.169), and the presence of psychiatric history (*β* = 0.217). In the R-UCLA model, higher loneliness scores were associated with higher depressive symptoms (*β* = 0.526), lower social network scores (*β* = −0.285), and older age (*β* = 0.148). We excluded sex, psychiatric history in the family, employment status, and receipt of social benefits from the final models due to a lack of statistically significant associations with either dependent variable.

Table 7 presents the regression coefficients and model fit indices for both models.

**Table 7:** Multiple linear regression for prediction of GHQ-28 and R-UCLA total scores in the total sample.

Independent variable	GHQ-28 total score			R-UCLA total score		
	B (SE)	*β*	*p*-value	B (SE)	*β*	*p*-value
Citizenship	6.735 (1.736)	0.225	**<0.001**	−0.360 (1.278)	−0.017	0.779
R-UCLA Total Score / GHQ-28 Total Score	0.734 (0.084)	0.516	**<0.001**	0.370 (0.043)	0.526	**<0.001**
C-SNI	0.082 (0.049)	0.103	0.094	−0.160 (0.033)	−0.285	**<0.001**
Age	−0.127 (0.042)	−0.169	**0.003**	0.078 (0.030)	0.148	**0.011**
Psychiatric history	8.262 (2.256)	0.217	**<0.001**	1.448 (1.651)	0.054	0.382
Model fit indices						
R^2^	0.403			0.391		
Adjusted R^2^	0.389			0.376		
ΔF (df1, df2), *p*-value	27.448 (5. 203), ***p* < 0.001**			26.116 (5. 203), ***p* < 0.001**		

SE, standard error; *p*, statistical significance; significant differences at *p* ≤ 0.05 are marked in bold.

## 4 Discussion

The objective of this study was threefold: (1) to compare levels of psychological distress, loneliness, and social network strength between Ukrainian refugees and Polish citizens in Wroclaw amidst the ongoing war and migration crisis; (2) to explore the relationships among psychological distress, loneliness, and social networks; and (3) to identify key sociodemographic and psychosocial factors associated with mental health and loneliness. As expected, Ukrainian refugees demonstrated higher levels of psychological distress compared to Polish citizens. In contrast, loneliness levels did not differ significantly between the two groups. Refugees, however, reported stronger social networks, particularly with extended family members. Psychological distress and depressive symptoms were associated with greater loneliness, while stronger social networks were linked to lower loneliness levels. Notably, 75% of Ukrainian refugees in our sample reported clinically relevant levels of psychological distress. This proportion aligns with findings from previous studies on Ukrainian refugees in other countries, where 64–74% of participants reported significant mental health concerns (45–49). Psychological distress among war migrants was most strongly expressed through somatic symptoms, anxiety, and insomnia, consistent with patterns reported in previous studies. Estimates from prior research suggest that approximately half of all refugees experience at least moderate levels of anxiety (5, 50–53). The elevated prevalence of depressive symptoms observed in war migrants, as measured by the GHQ-28, may reflect the life-threatening experiences they faced in their home country. One study found that higher levels of war exposure, such as witnessing bombings or active combat, were associated with reduced forgiveness, lower faith maturity, and increased anger toward God, all of which negatively affected psychological well-being (54). Ongoing psychological distress may also be intensified by persistent exposure to media coverage of military operations and social media content related to the war (55). Uncertainty regarding the duration of the conflict, prospects for returning home, and the safety of loved ones further contributes to elevated stress levels (56). Feelings of guilt may also emerge among those who found refuge in Poland while leaving family members behind. Notably, we observed no significant differences in severe depressive symptoms between Ukrainian war migrants and Polish citizens. This finding appears to contrast with previous studies reporting higher rates of depression among Ukrainian refugees (5, 52, 53). However, differences in measurement tools may help explain this discrepancy. For example, some studies have used instruments such as the PHQ-8 to assess depressive symptoms, which may capture a broader range or different severity levels than the GHQ-28 subscale applied in our study. Additionally, the war migrants in our sample were, on average, older than the Polish participants. Given that younger individuals in our study tended to report higher depressive symptom scores, this age difference may have influenced the results. Another noteworthy finding is the absence of significant gender differences in psychological distress and loneliness among Ukrainian refugees. This contrasts with several previous studies, which reported poorer mental health outcomes among Ukrainian women compared to men (5, 46, 52, 57). However, other research on Ukrainian civilians in conflict-affected areas or among internally displaced populations has also reported an absence of gender differences in psychological distress (7, 58). Ukrainian refugees had a higher unemployment rate compared to Polish citizens, which may be associated with increased psychological distress. This observation aligns with previous research linking financial hardship and unemployment to poorer mental health outcomes (6). Regarding loneliness, approximately half of Ukrainian refugees in our sample reported moderate to moderately high levels. This aligns with findings from other refugee studies, where reported rates of loneliness range from 16 to 61% (59, 60). However, no significant differences in loneliness levels were observed between Ukrainian war migrants and Polish citizens. One possible explanation is the hospitable reception that Ukrainian refugees received in Poland. This may be partially attributed to the linguistic and cultural proximity between the two nations, as well as the large Ukrainian diaspora already present in Poland prior to the war (61). In a survey conducted by Babicki et al. (62), nearly 80% of Polish respondents supported free access to medical care for refugees, and 85% endorsed free access to education for migrants. Previous studies suggest that perceived hospitableness in the host country can reduce feelings of loneliness and promote psychological adjustment (61, 63). Moreover, stronger identification with the host society has been linked to lower levels of post-traumatic stress and greater post-traumatic growth among refugees (64). Loneliness was inversely correlated with social network strength in our study, and war migrants demonstrated more developed social ties, particularly in the ‘other family members’ component of the CSNI. One possible explanation is the age distribution of the sample: Ukrainian participants were more often middle-aged, whereas Polish citizens included a larger proportion of younger individuals, especially students. Middle-aged adults are more likely to have established family networks, including children. In our sample, 76.5% of Ukrainian respondents reported having children, compared to 31.2% of Polish citizens. Additionally, many refugees fled with their families, for example, women crossing borders with their children (65, 66), which may have strengthened intra-familial bonds during the migration process. Previous research has shown that shared migration experiences can enhance family cohesion and relational closeness (67). Our study found a significant positive correlation between psychological distress, depressive symptoms, and loneliness, consistent with previous research in both general and refugee populations (40, 57). Belau et al. found that lower levels of social integration and support were associated with increased loneliness, which, in turn, negatively impacted mental health. Their findings also indicated that loneliness mediated the relationship between social integration and overall well-being among refugees in Germany (68). Similarly, among Ukrainian refugees relocated to Russia, loneliness was strongly associated with depression, low resilience, and reduced quality of life (69). It is important to note that the process of social integration may have negative aspects, such as exposure to hate speech, which can contribute to acculturation stress and deterioration of mental health (70). As demonstrated in our analysis, stronger social networks were associated with lower loneliness; however, no significant correlation was found between social networks and depressive symptoms, as measured by the GHQ-28. Previous research suggests that while social support can mitigate depressive symptoms, it may also increase exposure to war-related stress through conversations about traumatic experiences (57). At the same time, close family bonds have been identified as a protective factor that can enhance resilience among refugees (53). Although our study did not employ dedicated instruments for measuring social support or social capital, our findings can be partly interpreted within these theoretical frameworks. The CSNI index, used in this study, reflects the size, proximity, frequency, and emotional closeness of one’s social relationships - dimensions that align conceptually with structural social capital and forms of instrumental or companionship support (24, 30). Similarly, the R-UCLA loneliness scale, while primarily designed to assess subjective social isolation, includes items such as *“There is no one I can turn to”* or *“I feel left out,”* which overlap with perceived social support and thus can be viewed as indicators of cognitive social capital (23). In light of these connections, our findings suggest that Ukrainian refugees, who reported more developed social networks than Polish participants, may possess relatively stronger bonding social capital. Despite comparable levels of perceived loneliness between groups, psychological distress remained higher among refugees, which contrasts with prior findings suggesting a protective role of social support and social capital in displaced populations (20, 21, 71). One possible interpretation is that the presence of family and close social ties may reduce feelings of loneliness, but may not be sufficient to protect against broader psychological distress. The significant positive association between loneliness and psychological distress observed in our regression model supports the stress-buffering hypothesis, which posits that perceived social support can moderate the mental health impact of external stressors (19). At the same time, our findings raise the possibility that while bonding social capital was relatively strong among refugees, other dimensions such as bridging and linking capital were less available. Bridging capital refers to relationships between socially diverse individuals, and linking capital refers to connections with institutions and formal support structures. These components are often necessary for navigating bureaucratic systems, accessing health care, and engaging with the host society. Previous research has shown that the absence of these broader relational resources may limit the long-term mental health benefits of close interpersonal ties alone (23, 33, 72).

### 4.1 Practical and policy implications

Given the observed links between loneliness, social networks, and psychological distress, our results point to specific intervention priorities for supporting the mental health of Ukrainian war migrants. The strong association between loneliness and psychological distress suggests that reducing perceived social isolation may be a key target for improving well-being. At the same time, more developed social networks among refugees, particularly involving extended family, did not appear sufficient to mitigate psychological distress. This highlights that while bonding social capital may reduce loneliness, it does not necessarily protect against broader psychological consequences of displacement. Therefore, interventions should not only maintain existing social ties, but also enhance the perceived quality and supportiveness of those relationships, especially for individuals who may appear socially connected but still experience emotional distress. Zabłocka-Żytka et al. recommend actions such as disseminating reliable mental health information, improving access to diagnosis and treatment, and fostering cross-sectoral and institutional collaboration to support both refugees and those involved in their care (73). To provide effective support, mental health care for refugees should be embedded within a broader framework that addresses basic needs, ensures access to essential services, and reduces the risk of long-term psychological consequences. Establishing links with culturally and linguistically appropriate services across voluntary, charitable, and public sectors is essential (74, 75). Expanding access to basic mental health first aid by training non-medical personnel such as transport workers and caseworkers may help foster a supportive and trauma-informed environment. These individuals can also be trained to recognize common signs of distress requiring professional attention. Moreover, developing clear and accessible pathways to primary care and mental health services, including financial support where needed, is crucial for addressing war-related trauma among Ukrainian refugees (76). These actions correspond closely to the goals of social capital-based interventions, as described in prior studies (23, 30, 31). In particular, initiatives aimed at fostering trust, reciprocity, and social connectedness have been shown to reduce psychological distress, especially when they promote active participation in community life and meaningful interpersonal relationships. Unlike structural reforms alone, these approaches address the relational dimension of refugee well-being by supporting both bonding ties within families and communities, and bridging and linking connections with the host society and public institutions. Interventions such as peer support programs, culturally adapted group activities, and refugee-led networks may be particularly effective in enhancing social participation and resilience. Coordinated efforts between municipalities, NGOs, and grassroots actors can facilitate not only access to services but also the sense of belonging and agency that underpins cognitive and structural social capital. By focusing on the quality of social relationships and inclusion in communal life, these strategies complement formal systems of care and contribute to long-term psychological recovery. However, recent studies have highlighted a persistent mismatch between the needs of refugees and the actual support provided in Poland, both in the short and long term (77). Two major barriers to effective healthcare delivery include the lack of prior medical documentation and significant language obstacles, which complicate communication between refugees and medical personnel (78). While targeted systemic solutions could reduce the daily workload of healthcare providers, no comprehensive governmental strategy has been implemented to ensure sustainable support or integration. As a result, the prolonged inadequacy of state-level assistance contributes to negative social attitudes and growing tensions between host communities and refugee populations (77, 78).

### 4.2 Limitations

Several limitations should be considered when interpreting the findings of this study. First, the cross-sectional design limits the ability to draw causal inferences or assess changes over time. Mental health status, loneliness, and social network structure are likely to evolve depending on factors such as length of stay and stage of integration. Longitudinal research would provide a more comprehensive understanding of these dynamics. Second, the study relied on self-reported measures of psychological distress, loneliness, and social network characteristics, which may be subject to social desirability or recall bias. Additionally, the Ukrainian version of the survey was self-translated. Although care was taken to ensure linguistic accuracy, replication in other Ukrainian samples using validated instruments is needed to confirm the reliability and clarity of the measures.

Third, the sampling method may have introduced bias in terms of representativeness. Due to the character of the project, which focused on direct engagement with participants, building interpersonal contact, and including individuals who might be excluded from online studies, we employed a convenience sampling strategy. Participants were recruited in public spaces and refugee assistance centers across Wroclaw, enabling real-life data collection. Although this approach aligned with the project’s goals and allowed for the inclusion of vulnerable populations, it may have limited sample diversity and generalizability. However, several measures were taken to reduce sampling bias, such as deliberately approaching individuals with diverse visible characteristics and regularly changing the interviewers’ positions within each site. Future studies should consider using randomized or stratified sampling techniques to further enhance representativeness and external validity.

Fourth, while elements of our results were discussed in light of the stress-buffering hypothesis and social capital theory, this was an exploratory, interpretative approach rather than a primary objective of the study. Our analyses were not based on instruments specifically designed to assess these theoretical frameworks. Although the CSNI and R-UCLA scales provide relevant insight into social connectedness and perceived support, future studies should incorporate validated tools that directly measure different dimensions of social capital and stress-buffering mechanisms. This would enable more precise theoretical testing and facilitate cross-study comparison.

Fifth, although several sociodemographic and clinical variables such as education level, receipt of social benefits, and personal or family history of psychiatric disorders significantly differed between the two groups, they did not show statistically significant associations with psychological distress or loneliness in our multiple regression models and were therefore excluded. Nevertheless, these between-group differences may have introduced residual confounding that could affect the results obtained from psychometric scales. For example, although personal psychiatric history was significantly associated with psychological distress in the final regression model, it was reported much less frequently by Ukrainian refugees than by Polish citizens. This discrepancy may reflect underdiagnosis, stigma, or limited prior access to mental health care. As a result, the observed group difference in psychological distress may underestimate the true burden among refugees and should be interpreted with caution.

Finally, the total sample size in our study was relatively small (*N* = 209), consisting of 68 Ukrainian refugees and 141 Polish citizens. Although this number allowed for valid statistical analyses, including regression models, it may limit the detection of more subtle effects and reduce generalizability. One of the reasons for the modest sample size was our methodological decision to limit recruitment to in-person, field-based assessments using paper-and-pencil questionnaires. This approach was not incidental but stemmed from the specific character of the broader project, which aimed not only to collect data but also to foster human connection, establish trust, and promote engagement, particularly with vulnerable populations such as refugees. By conducting surveys in person, we sought to reduce barriers to participation and create an inclusive environment in which participants could ask questions and receive clarification when needed. Establishing direct interpersonal contact was a deliberate element of our research design. Compared to online surveys, in-person data collection may offer several advantages. It allows for better sampling control, improves comprehension of questionnaire items, enhances participant motivation, and reduces the risk of inattentive or random responses. It also enables the inclusion of participants who might be excluded from online research due to lack of internet access, digital literacy, or technological trust. This is especially relevant in refugee populations, who often experience digital exclusion. Moreover, the presence of the researcher fosters engagement and allows for contextual observations that enrich data quality. These strengths have been emphasized in a previous analysis comparing field-based and online studies (79). Although our strategy may have contributed to a smaller sample size and reduced scalability, it helped ensure higher data reliability and greater inclusivity. Future studies may consider a mixed-mode approach to balance reach and data quality.

## 5 Conclusion

This study confirms that Ukrainian war refugees experienced higher levels of psychological distress than Polish citizens, likely due to war-related trauma. Psychological distress among refugees was primarily associated with somatic symptoms, anxiety, and insomnia, while levels of depressive symptoms were comparable between the two groups. Loneliness levels did not differ significantly, which may be attributed to the refugees’ stronger social networks and Poland’s relatively welcoming reception. A positive association between loneliness and mental health problems was also observed. These findings underscore the need for culturally and linguistically appropriate mental health interventions, including the dissemination of reliable information about mental health, accessible psychological services, and basic mental health first aid. State-level coordination is essential to ensure that such support measures are implemented effectively and sustainably. Despite its contributions, this study has several limitations, including its cross-sectional design, reliance on self-reported measures, relatively small sample, non-random sampling, and the potential influence of unmeasured or residual confounding factors. Future longitudinal research with more representative samples is needed to better understand the evolving mental health and social integration of Ukrainian refugees and Polish citizens.

## Data Availability

The raw data supporting the conclusions of this article will be made available by the authors, without undue reservation.

## Footnotes

^1^ www.r-project.org
