# Supplementary material for: Comparison of psychological distress, loneliness, and social network structure between Ukrainian war refugees and Polish citizens: a cross-sectional study in Wroclaw
Source: Front Public Health. 2025 Jul 15;13:1621003. doi: 10.3389/fpubh.2025.1621003 (PMC12303934; doi:10.3389/fpubh.2025.1621003)
Supplement: Supplementary file 1 [file Table_1.DOCX]

Supplementary Material

# Supplementary Tables

**Table S1. Spearman’s rho Correlation Matrix for Age and Psychometric Scale Scores in Ukrainian Refugees (N = 68)**

| **Parameter** | **Age** | **GHQ-28 Total Score** | **Somatic Symptoms** | **Anxiety /Insomnia** | **Social Dysfunction** | **Severe Depression** | **R-UCLA Total Score** | **Intimate Others** | **Social Others** | **Belonging and Affiliation** | **C-SNI** | **Spouse /Partner** | **Parents** | **Other Family Members** | **Neighbours** |
| --- | --- | --- | --- | --- | --- | --- | --- | --- | --- | --- | --- | --- | --- | --- | --- |
| **GHQ-28 Total Score** | -0,005 |  |  |  |  |  |  |  |  |  |  |  |  |  |  |
| Somatic Symptoms | 0,059 | **,748**** |  |  |  |  |  |  |  |  |  |  |  |  |  |
| Anxiety/Insomnia | -0,003 | **,873**** | **,594**** |  |  |  |  |  |  |  |  |  |  |  |  |
| Social Dysfunction | 0,031 | **,783**** | **,394**** | **,689**** |  |  |  |  |  |  |  |  |  |  |  |
| Severe Depression | -0,203 | **,604**** | **,262*** | **,411**** | **,439**** |  |  |  |  |  |  |  |  |  |  |
| **R-UCLA Total Score** | -0,003 | **,593**** | **,298*** | **,555**** | **,515^**^** | **,666**** |  |  |  |  |  |  |  |  |  |
| Intimate Others | 0,023 | **,614**** | **,335**** | **,593**** | **,519**** | **,603**** | **,939**** |  |  |  |  |  |  |  |  |
| Social Others | -0,109 | **,487**** | **,240*** | **,455**** | **,382**** | **,616**** | **,856**** | **,714**** |  |  |  |  |  |  |  |
| Belonging and Affiliation | 0,028 | **,443**** | 0,2 | **,351**** | **,412**** | **,584**** | **,824**** | **,643**** | **,706**** |  |  |  |  |  |  |
| **C-SNI** | 0,096 | -0,188 | -0,059 | -0,169 | -0,153 | **-,302*** | **-,363**** | **-,309*** | **-0,403** | **-,340**** |  |  |  |  |  |
| Spouse/Partner | -0,018 | -0,234 | -0,142 | **-,240*** | -0,213 | **-,251*** | **-,294*** | **-,273*** | **-,331**** | -0,227 | **,867**** |  |  |  |  |
| Parents | **-,561**** | -0,197 | -0,196 | -0,142 | -0,137 | -0,003 | -0,146 | -0,173 | -0,127 | -0,112 | 0,113 | 0,023 |  |  |  |
| Other Family Members | **,620**** | 0,029 | 0,091 | 0,093 | 0,073 | **-,249*** | -0,132 | -0,048 | -0,232 | -0,169 | **,491**** | 0,23 | **-,334**** |  |  |
| Neighbours | -0,012 | -0,148 | 0,03 | -0,138 | -0,131 | **-,300*** | **-,374**** | **-,315**** | **-,380**** | **-,403**** | **,491**** | **,288*** | **,246*** | 0,113 |  |
| Friends and Co-workers | -0,003 | **-,405**** | -,324** | **-,320**** | **-,320**** | **-,273*** | **-,420**** | **-,418**** | **-,451**** | **-,322**** | **,514**** | **,373**** | **,288*** | 0,075 | **,416**** |
| The values in the table are correlation coefficients; significant are marked in bold; *. Correlation is significant at the 0.05 level (2-tailed); **. Correlation is significant at the 0.01 level (2-tailed). | | | | | | | | | | | | | |  |  |

**Table S2. Spearman’s rho Correlation Matrix for Age and Psychometric Scale Scores in Polish Citizens of Wroclaw (N = 141)**

| **Parameter** | **Age** | **GHQ-28 Total Score** | **Somatic Symptoms** | **Anxiety /Insomnia** | **Social Dysfunction** | **Severe Depression** | **R-UCLA Total Score** | **Intimate Others** | **Social Others** | **Belonging and Affiliation** | **C-SNI** | **Spouse /Partner** | **Parents** | **Other Family Members** | | **Neighbours** |
| --- | --- | --- | --- | --- | --- | --- | --- | --- | --- | --- | --- | --- | --- | --- | --- | --- |
| **GHQ-28 Total Score** | **-,252**** |  |  |  |  |  |  |  |  |  |  |  |  |  | |  |
| Somatic Symptoms | **-,216*** | **,793**** |  |  |  |  |  |  |  |  |  |  |  |  | |  |
| Anxiety/Insomnia | **-,231**** | **,837**** | **,610**** |  |  |  |  |  |  |  |  |  |  |  | |  |
| Social Dysfunction | -0,126 | **,739**** | **,458**** | **,499**** |  |  |  |  |  |  |  |  |  |  | |  |
| Severe Depression | **-,183*** | **,750**** | **,403**** | **,551**** | **,535**** |  |  |  |  |  |  |  |  |  | |  |
| **R-UCLA Total Score** | -0,021 | **,491**** | **,324**** | **,391**** | **,373**** | **,520**** |  |  |  |  |  |  |  |  | |  |
| Intimate Others | -0,007 | **,480**** | **,334**** | **,422**** | **,341**** | **,529**** | **,916**** |  |  |  |  |  |  |  | |  |
| Social Others | -0,099 | **,385**** | **,246**** | **,293**** | **,324**** | **,334**** | **,848**** | **,687**** |  |  |  |  |  |  | |  |
| Belonging and Affiliation | -0,003 | **,375**** | **,227**** | **,267**** | **,282**** | **,383**** | **,748**** | **,501**** | **,594**** |  |  |  |  |  | |  |
| **C-SNI** | **,327**** | -0,12 | -0,038 | -0,09 | **-,185*** | -0,127 | **-,304**** | **,308**** | **-,354**** | -0,105 |  |  |  |  | |  |
| Spouse/Partner | 0,019 | -0,033 | 0,009 | 0,029 | -0,160 | -0,104 | **-,278**** | **-,301**** | **-,298**** | -0,071 | 0,824 |  |  |  | |  |
| Parents | **-,550**** | 0,082 | 0,108 | 0,042 | 0,004 | 0,006 | -0,134 | -0,158 | 0,009 | -0,101 | 0,054 | 0,162 |  |  | |  |
| Other Family Members | **,553**** | -0,126 | -0,021 | **-,185*** | -0,045 | -0,007 | 0,033 | 0,049 | -0,095 | 0,026 | **,352**** | -0,076 | **-,405**** |  | |  |
| Neighbours | **,396**** | **-,204*** | **-,171*** | -0,172 | **-,220**** | -0,145 | -0,094 | -0,024 | -0,157 | -0,107 | **,322**** | -0,038 | **-,186*** | **,400**** | |  |
| Friends and Co-workers | -0,124 | -0,052 | 0,036 | -0,052 | -0,053 | -0,136 | **-,320**** | **-,313**** | **-,210*** | **-,177*** | **,194*** | 0,069 | **,313**** | -0,126 | | 0,146 |
| The values in the table are correlation coefficients; significant are marked in bold; *. Correlation is significant at the 0.05 level (2-tailed); **. Correlation is significant at the 0.01 level (2-tailed). | | | | | | | | | | | | | | |  |  |
